# Associations between pre-pregnancy psychosocial risk factors and infant outcomes: a population-based cohort study in England

**DOI:** 10.1016/S2468-2667(20)30210-3

**Published:** 2021-01-28

**Authors:** Katie Harron, Ruth Gilbert, Jamie Fagg, Astrid Guttmann, Jan van der Meulen

**Affiliations:** a UCL Great Ormond Street Institute of Child Health, London, UK; b Imperial College NHS Foundation Trust, St Mary's Hospital, London, UK; c IC/ES, Toronto, ON, Canada; d London School of Hygiene & Tropical Medicine, London, UK

## Abstract

**Background:**

Existing studies evaluating the association between maternal risk factors and specific infant outcomes such as birthweight, injury admissions, and mortality have mostly focused on single risk factors. We aimed to identify routinely recorded psychosocial characteristics of pregnant women most at risk of adverse infant outcomes to inform targeting of early intervention.

**Methods:**

We created a cohort using administrative hospital data (Hospital Episode Statistics) for all births to mothers aged 15–44 years in England, UK, who gave birth on or after April 1, 2010, and who were discharged before or on March 31, 2015. We used generalised linear models to evaluate associations between psychosocial risk factors recorded in hospital records in the 2 years before the 20th week of pregnancy (ie, teenage motherhood, deprivation, pre-pregnancy hospital admissions for mental health or behavioural conditions, and pre-pregnancy hospital admissions for adversity, including drug or alcohol abuse, violence, and self-harm) and infant outcomes (ie, birthweight, unplanned admission for injury, or death from any cause, within 12 months from postnatal discharge).

**Findings:**

Of 2 520 501 births initially assessed, 2 137 103 were eligible and were included in the birth outcome analysis. Among the eligible births, 93 279 (4·4%) were births to teenage mothers (age <20 years), 168 186 (7·9%) were births to previous teenage mothers, 51 312 (2·4%) were births to mothers who had a history of hospital admissions for mental health or behavioural conditions, 58 107 (2·7%) were births to mothers who had a history of hospital admissions for adversity, and 580 631 (27·2%) were births to mothers living in areas of high deprivation. 1 377 706 (64·5%) of births were to mothers with none of the above risk factors. Infants born to mothers with any of these risk factors had poorer outcomes than those born to mothers without these risk factors. Those born to mothers with a history of mental health or behavioural conditions were 124 g lighter (95% CI 114–134 g) than those born to mothers without these conditions. For teenage mothers compared with older mothers, 3·6% (95% CI 3·3–3·9%) more infants had an unplanned admission for injury, and there were 10·2 (95% CI 7·5–12·9) more deaths per 10 000 infants.

**Interpretation:**

Health-care services should respond proactively to pre-pregnancy psychosocial risk factors. Our study demonstrates a need for effective interventions before, during, and after pregnancy to reduce the downstream burden on health services and prevent long-term adverse effects for children.

**Funding:**

Wellcome Trust.

## Introduction

Psychosocial factors in pregnant women that are not addressed can have adverse effects on outcomes of pregnancy for both mothers and their infants.^1^ Guidelines from the National Institute for Health and Care Excellence recommend that women with “complex social risk factors” should be identified during pregnancy so that additional support can be provided.^2^ These guidelines recognise that vulnerable women often experience a range of social risk factors simultaneously, and highlight the specific needs of teenage mothers (aged <20 years), women who misuse substances, and women who experience domestic violence.

Targeted support for women before and during pregnancy has the potential to improve outcomes at and after birth, through promoting preconception health and reproductive choices and mitigating the adverse effects of maternal stress.^3^, ^4^, ^5^ Postnatal support can positively affect the quality of caregiving and child attachment, and the development and behaviour of the child; furthermore, it can mitigate the effects of adversity and reduce the risk of unmet medical need or child injury.^6^ Appropriate early intervention can therefore lead to improved maternal and neonatal outcomes, and health, education, and social outcomes throughout childhood.^7^

In England, maternity services and primary care during and after pregnancy are universal and freely available for the approximately 700 000 births per year, with home visiting support delivered through the Healthy Child Programme.^8^ The frequency of home visits and contact varies according to a proportionate universalism model, meaning that whereas support is universal, scale and intensity is proportionate to the level of need and disadvantage.^8^, ^9^ In practice, groups who stand to benefit from additional support (ie, for whom early intervention might be effective) are identified as those likely to experience adverse health outcomes. Previous evidence has identified associations between poor birth and infant outcomes and risk factors that are routinely recorded in hospital records such as young maternal age, parity, risky behaviours (including smoking, drug or alcohol misuse, or poor diet), exposure to intimate partner violence, maternal mental health, and poor engagement with antenatal care services.^5^, ^10^, ^11^, ^12^, ^13^, ^14^, ^15^, ^16^ Routine hospital records can be used to identify psychosocial risk factors and their influence on child outcomes.^11^, ^17^, ^18^, ^19^ However, most previous studies focused on single risk factors, and there is a gap in the evidence on which of these risk factors—when considered together—are associated with the highest risk. There is an absence of studies using routine records to explore multiple psychosocial risk factors across maternal age groups, before and during early pregnancy.

**Research in context**

**Evidence before this study**

We searched PubMed for studies published in English between Jan 1, 2000, and May 1, 2020, using the MeSH terms “pregnancy outcome”, birthweight“, “infant mortality”, OR “infant, newborn”, AND “maternal age”, “adolescence”, “parity”, “social class”, “substance-related disorders” “violence”, OR “mental disorders”. We reviewed studies from high-income countries that addressed maternal risk factors before and during pregnancy in relation to birth outcomes, or infant hospital admissions or mortality. Previous cohort and administrative data longitudinal studies have examined the association between young maternal age and parity and specific maternal or infant health outcomes, and they have found that young maternal age is associated with low birthweight, preterm birth, and increased health-care use throughout childhood. Population-based cohorts also report links between poor maternal mental health during pregnancy and low birthweight or preterm birth, and between poor maternal mental health during pregnancy and injury and respiratory symptoms in children. Other studies have found increased risks of infant morbidity and mortality or poor child development associated with risky behaviours of the mother recorded during pregnancy (including smoking, drug or alcohol misuse, or poor diet), mother exposure to intimate partner violence, maternal mental health, or lack of antenatal care. Many studies have also highlighted disparities for those with lower versus higher social status, and differences in risk of adverse infant and child health outcomes by ethnicity, race, or Aboriginal status. Most studies focused on single risk factors; we did not find any studies using population-based administrative data to quantify the association of multiple psychosocial risk factors that are routinely recorded by health services before or during pregnancy, and infant birthweight, injury admissions, and mortality.

**Added value of this study**

This population-based cohort study examined data from more than 2 million mother–baby pairs in National Health Service hospitals in England, UK, over 5 years. This study showed that pre-pregnancy psychosocial risk factors routinely recorded in hospitalisation records before 20 weeks of pregnancy (ie, previous birth before 20 years of age, hospital contacts related to adversity or mental health or behavioural conditions, and deprivation) were associated with substantially increased risks of low birthweight, preterm birth, injury, and death during the 12 months from postnatal discharge. Maternal age of less than 20 years for the current or previous birth was an important risk factor, but hospitalisations before pregnancy for drug or alcohol abuse, self-harm, or violence, or for mental health or behavioural conditions were also important, irrespective of maternal age. These groups could potentially benefit from health and social interventions before and during pregnancy.

**Implications of all the available evidence**

This research shows a need for effective interventions before, during, and after pregnancy to reduce the downstream burden on health services and prevent long-term adverse effects for children, including low birthweight, unplanned admission for injury, and mortality. In addition to the 11·3% of mothers who were teenagers at the current or a previous birth, we found that the 4% of women aged 20–44 years who could be routinely identified from hospital data before pregnancy as having a history of admission to hospital for adversity or mental health or behavioural conditions also had poor birth and infant outcomes, as did those living in the most deprived population quintile. Improved data collection, sharing, and linkage across multiple data sources, and efforts across primary and secondary care to respond to psychosocial risk factors in women using health care (especially among maternity and health visiting services) could improve support for women before, during, and after pregnancy, and potentially reduce adverse outcomes. More research is needed to develop effective interventions for women with different risk factors. Given the disparities in outcomes across quintiles of deprivation (ie, poorer outcomes for the 27% of births to women living in the most deprived areas), strategies to address the root causes of social disadvantage are also required. Our findings apply to England, but could be generalisable to other countries with similar maternal risk factors.

Understanding which women are most vulnerable, and how to identify them in time for intervention, is the first step to the effective development and targeting of early health and social support programmes.^20^ This study used population-based hospital data to evaluate the association between multiple maternal psychosocial risk factors that can be identified routinely and early in pregnancy (teenage motherhood, a history of hospital admissions for adversity or mental health conditions, or deprivation) and key birth and infant outcomes (birthweight, unplanned hospital admissions, and mortality) that might be amenable to intervention. Our aim is to inform clinical and political decision making on early targeting of health and social support before, during, and after pregnancy.

## Methods

### Study design and participants

This study is a population-based cohort study in England. Hospital records were obtained from Hospital Episode Statistics (HES), an administrative database holding detailed information for all admissions to the National Health Service hospitals in England.^21^ Admission records allow the entry of up to 20 fields of clinical diagnoses coded using the International Statistical Classification of Diseases and Related Health Problems 10th Revision (ICD-10).

The study population consisted of a cohort of mother–baby pairs, which was created by probabilistic linkage of deliveries and livebirths within HES.^22^ We included mother–baby pairs when births were singleton births, mothers were aged 15–44 years at delivery, delivery occurred on or after April 1, 2010, and discharge happened before and on March 31, 2015. We excluded the small number of births to mothers aged 45 years and older, because adverse outcomes for this group might relate to biological age (rather than the social risk factors) and these age-related risk factors are less likely to be amenable to intervention. We excluded births to mothers younger than 15 years because of the small numbers. For mothers with multiple deliveries during the study period, we used a random number generator to select one mother–baby pair for inclusion in analyses, to avoid clustering of outcomes within mothers. We did not restrict analyses to the first child, so that we could consider parity, and so that our findings would be more widely generalisable. Approvals for the use of HES data were obtained as part of the standard Hospital Episode Statistics approval process and ethical approval was obtained from London—South East Research Ethics Committee (reference 16/LO/0012). HES records were made available by NHS Digital.

### Outcomes

We chose outcomes that might be amenable to support for women before, during, or after pregnancy and that align with the priorities of English National Health Service antenatal care and the Healthy Child Programme.^8^, ^23^ First, we described birthweight, because low birthweight is a predictor of substantial morbidity. Birthweight was modelled as a continuous variable, and babies born weighing less than 2500 g were categorised as having low birthweight. We also described gestational age (based on best estimates from menstrual dates or ultrasound) and size for gestation at birth. These variables were derived from maternity fields in infant or mother HES records.^22^ We then evaluated the number of infants with one or more unplanned hospital admissions for injury within 12 months from postnatal discharge. Reducing hospital admissions and accidents is a high impact area of the Healthy Child Programme, and th come is considered to be amenable to change through postnatal support delivered by health visitors.^24^ Admissions were defined as unplanned on the basis of the method of admission code (ie, excluding elective admissions), and comprised episodes of care in any hospital captured within the HES data, starting more than 2 days after the end of the birth episode. We used published lists of ICD-10 diagnosis codes to identify admissions for injury (appendix pp 3–5).^25^ Finally, we measured infant mortality in the 12 months following postnatal discharge (referred to as post-discharge mortality). We evaluated these infant outcomes from postnatal discharge, rather than birth, to reflect events at home in the care of parents and to allow for different lengths of stay during the birth or delivery admission.

### Risk factors

We pre-specified a number of psychosocial risk factors on the basis of the guidelines for antenatal care for women with complex social needs, namely: teenage motherhood (ie, mothers aged 15–19 years at delivery), previous teenage motherhood (ie, mothers aged 20 years or older at delivery, with a previous birth when aged 15–19 years), history of adversity-related admissions (for violence, substance misuse, or self-harm), history of admissions for mental health or behavioural conditions (excluding self-harm), and living in areas of high deprivation.

To define histories of adversity or histories of mental health or behavioural conditions, we examined hospital admission records in the 2 years before the mothers’ 20th week of pregnancy. We chose 20 weeks on the basis of recommendations that nearly all women have their first antenatal contact (usually a booking appointment) before this time. Guidelines recommend that women are examined by 10 weeks of pregnancy, but the first examination is often later for women with risk factors; approximately 90% of all women are seen by 20 weeks of gestation.^26^ Although recording of these risk factors might also occur after 20 weeks of pregnancy, we aimed to define risk factors that could be identified early in pregnancy. We used a 2-year look-back period for hospital admissions, because this period before birth is likely to have effects on outcomes and should be in the patient records or could be asked about by clinicians. We also examined the number of women identified as having a history of mental health conditions or adversity within 1 year and within 5 years before the 20 weeks of pregnancy. We identified previous admissions for adversity (violence, substance misuse, self-harm), or mental health or behavioural conditions, on the basis of published lists of ICD-10 diagnosis codes (appendix pp 3–5).^25^, ^27^ We chose to categorise self-harm within the adversity category because it is a behaviour or event (which can be a manifestation of a mental health condition), and to align with previous literature.^25^

Quintiles of socioeconomic deprivation were derived from the national distribution of the Index of Multiple Deprivation 2004 on the basis of the postcode of residence at delivery.^28^ Areas of high deprivation were defined as those within the most deprived quintile. We also described parity and ethnicity. Mothers were classified as primiparous if the number of previous pregnancies recorded on the maternity record was zero, unless we found a previous delivery for the mother present in HES records from 2000 onwards. Previous teenage birth was defined according to age at first birth recorded in HES; a small proportion of mothers might have been misclassified if they had their first birth before 2000 (ie, >10 years before the study period), or before entering the HES cohort (eg, immigrant mothers). Ethnic group was derived from the maternal record.

### Statistical analysis

Because this study was a population-based study, no sample size calculation was done. We first calculated the number of women experiencing each risk factor, and the association between risk factors and maternal age group. We then used generalised linear models to estimate the association between individual year of maternal age, psychosocial risk factors, and outcomes, using Schoenberg B-splines for age to allow for a non-linear effect.^29^ This approach is based on a set of reference points or knots, with the number of points chosen to balance smoothness and goodness of fit. To avoid overfitting in such a large study cohort, we present results from models with a minimal number of a-priori reference points (at ages 20, 25, 30, and 35 years). Results are presented graphically with interactions for each maternal risk factor. These plots do not present mortality and previous teenage birth, because of the low numbers. For unplanned admissions for injury and post-discharge infant mortality (but not birthweight), we excluded mother–baby pairs in which the baby would not have been exposed to the mother after the birth episode, ie, those discharged to social services or not surviving to postnatal discharge (appendix p 1).

To quantify the risk of adverse outcomes for women with each psychosocial risk factor, we derived risk differences from generalised linear models with a log link and relative risks from generalised linear models with an identity link, comparing women with each risk factor to women without that risk factor. We derived adjusted estimates to help understand how much of the associations were explained by concurrent risk factors. Adjusted models for birthweight included all psychosocial risk factors defined previously, plus maternal age (15–19, 20–24, 25–29, 30–34, 35–44 years, whereby the 35–44-year age group combines the two 5-year groups because of the low numbers), ethnicity, and parity. Models for unplanned admission for injury counted infants who had died from any cause after postnatal discharge as having the outcome (because death was a competing risk for admission). All models used robust standard errors to allow for clustering of women within hospitals. On the basis of these adjusted models, we derived population attributable fractions (PAFs), to quantify the percentage of outcomes in the study population attributable to each risk factor.

We used multiple imputation with chained equations for missing values of birthweight (6·6%), gestational age (7·5%), and deprivation (0·8%). The imputation models included birthweight, gestational age, quintile of deprivation, delivery by caesarean section, pregnancy or delivery and neonatal risk factors identified from ICD-10 codes (appendix p 6), ethnic group, parity, infant sex, maternal age, and psychosocial risk factors (previous teenage motherhood, history of adversity, history of mental health or behavioural conditions); we used five imputations. A complete case analysis was done as a sensitivity analysis. In both analyses, unknown or missing ethnicity was treated as a separate category. Analyses were done in Stata 15.

### Role of the funding source

The funder of the study had no role in study design, data collection, data analysis, data interpretation, or writing of the report. The corresponding author had full access to all the data in the study and had final responsibility for the decision to submit for publication.

## Results

Of 2 520 521 mother–baby pairs initially assessed for eligibility, 383 418 were excluded (appendix p 1). The study cohort consisted of 2 137 103 births occurring on or after April 1, 2010, with infants discharged before or at March 31, 2015 (appendix p 1). 299 526 (12·3%) mothers had multiple deliveries during the study period, for each of whom we selected one mother–baby pair for inclusion in the analyses. The prevalence of risk factors by age group is shown in the appendix (p 7). 759 397 (35·5%) mothers had at least one risk factor (14·3% when excluding those whose only risk factor was living in the most deprived areas; appendix p 7). Of the 58 107 (2·7%) mothers with a history of adversity in the 2 years before 20 weeks of pregnancy, 49 755 (85·6%) had admissions for substance misuse, 21 720 (37·4%) had admissions for self-harm, and 3520 (6·1%) had admissions for violence.

When looking back at the 5 years preceding the 20th week of pregnancy, we observed that 88 923 (4·2%) women had a history of mental health or behavioural conditions and 117 416 (5·5%) had a history of adversity. When looking at the 1 year preceding the 20 weeks of pregnancy, this number decreased to 26 967 (1·3%) for mental health or behavioural conditions and to 29 049 (1·4%) for adversity (appendix p 8).

The proportion of mothers with psychosocial risk factors tended to decrease with maternal age (appendix p 2). Having multiple risk factors or at least one risk factor was most common in teenage mothers and decreased with maternal age (appendix p 7).

**Figure 1:**
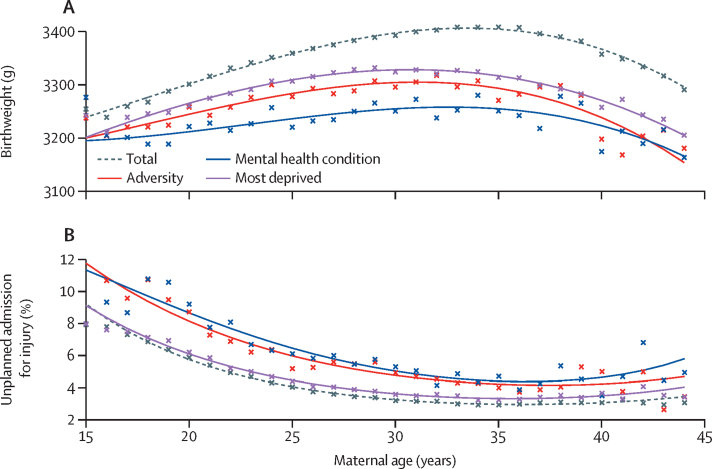
Association between maternal age and birth and infant outcomes (A) Crude (unadjusted) association between maternal age and birthweight. (B) Crude (unadjusted) association between maternal age and percentage of infants with one or more unplanned admissions for injury in the 12 months from postnatal discharge.

**Figure 2:**
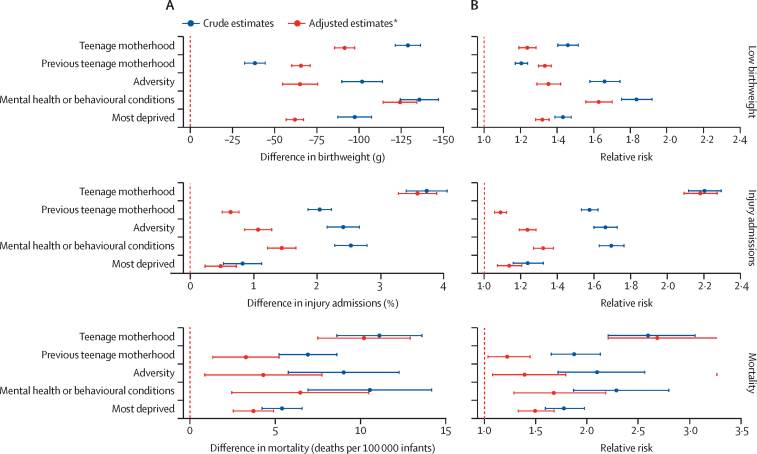
Comparisons of mothers with each risk factor versus mothers without that risk factor Crude and adjusted risk differences (A) and risk ratios (B) with 95% confidence intervals for low birthweight (<2500 g), unplanned admission for injury in the 12 months from postnatal discharge, and post-discharge infant mortality, according to psychosocial risk factor identified in the 2 years before 20 weeks of pregnancy (teenage motherhood, previous teenage motherhood, a history of adversity, a history of mental health or behavioural conditions, or living in the most deprived quintile according to the Index of Multiple Deprivation 2004). Comparisons are between mothers with each risk factor and mothers without that specific risk factor. *Adjusted for all psychosocial risk factors, ethnic group, and parity.

Babies born to the youngest or oldest mothers had the lowest birthweights, while the highest birthweights were observed for babies born to mothers aged 30–37 years (figure 1). At all maternal ages, babies born to mothers with any of the risk factors considered here tended to have lower birthweight compared to the average of the study population. A history of mental health or behavioural conditions was associated with the lowest birthweights comparatively (figure 1). Adjusting for multiple maternal risk factors attenuated effect sizes; eg, adjusting for current teenage motherhood attenuated the effect of adversity (data not shown). After adjustment, babies born to mothers with a history of mental health or behavioural conditions had the lowest birthweights, corresponding to a difference of 124 g (95% CI 114–134) compared with mothers without this risk factor (figure 2; appendix p 9). For these mothers, the adjusted relative risk for low birthweight was 1·63 (95% CI 1·56–1·70; appendix p 9). The highest PAF for low birthweight was for women living in the most deprived areas: 8·2% (95% CI 7·3–9·1) of low birthweights were attributable to deprivation (table 1).

**Table 1:** Adjusted population attributable fractions for low birthweight and unplanned admission for injury and mortality within the 12 months from postnatal discharge, according to psychosocial risk factors

	**Low birthweight (<2500 g)**	**One or more unplanned admissions for injury**	**Post-discharge mortality (deaths per 10 000 infants)**
Teenage motherhood	0·9% (0·8–1·1)	3·4% (3·2–3·7)	5·3% (3·9–6·6)
Previous teenage motherhood	2·1% (1·8–2·3)	2·9% (2·6–3·2)	3·7% (1·7–5·7)
History of adversity	1·1% (0·9–1·3)	0·9% (0·7–1·1)	1·4% (0·2–2·7)
History of mental health or behavioural conditions	1·7% (1·5–1·8)	1·0% (0·8–1·1)	1·9% (0·7–3·2)
Most deprivation*	8·2% (7·3–9·1)	5·0% (3·1–6·9)	13·5% (9·4–17·5)

Data are population attributable fraction (95% CI). Population attributable fractions were adjusted for all psychosocial risk factors, maternal age, ethnic group, and parity. Psychosocial risk factors were identified in the 2 years before 20 weeks of pregnancy.

* Most deprived quintile of the Index of Multiple Deprivation.

The percentage of infants with an unplanned admission for injury decreased with increasing maternal age, and was lowest for mothers aged 35–40 years (figure 1). The highest rates of unplanned admission for injury were seen in infants born to teenage mothers (table 2) and to mothers with a history of mental health conditions, across the spectrum of maternal age (figure 1). Despite the clustering of psychosocial risk factors in teenage mothers, this association remained after adjusting for other risk factors, corresponding to an additional 3·6% (95% CI 3·3–3·9%) of infants with unplanned admissions compared with mothers aged 20–44 years, or a relative risk of 2·18 (95% CI 2·09–2·27; figure 2, appendix p 9). The highest PAF for injury admissions was for women living in the most deprived areas: 5·0% (95% CI 3·1–6·9) of unplanned admissions for injury were attributable to deprivation (table 1).

**Table 2:** Crude (unadjusted) birth outcomes and infant outcomes within 12 months of postnatal discharge according to psychosocial risk factors

	**Birth outcomes**						**Infant outcomes within 12 months of postnatal discharge**		
	All birth outcomes	Mean birthweight (g)	Low birthweight (<2500 g)	Preterm birth (<37 weeks)	Small for gestation*	Large for gestation*	All infant outcomes within 12 months of postnatal discharge	One or more unplanned admissions for injury	Post-discharge mortality (deaths per 10 000 infants)
Total	2 137 103 (100·0%)	3374 (3373–3375)	5·6% (5·5–5·6%)	6·4% (6·4–6·4%)	7·1% (7·1–7·2%)	10·5% (10·5–10·6%)	2 129 227 (99·6%)	3·6% (3·6–3·7%)	8·4 (8·1–8·8)
Teenage motherhood	93 279 (4·4%)	3272 (3269–3276)	7·2% (7·0–7·4%)	7·4% (7·2–7·6%)	10·1% (9·9–10·3%)	6·6% (6·4–6·8%)	92 474 (4·3%)	6·6% (6·5–6·8%)	18·1 (15·4–21·0)
Previous teenage motherhood	168 186 (7·9%)	3338 (3335–3340)	6·5% (6·3–6·6%)	7·5% (7·5–7·7%)	7·1% (7·0–7·3%)	10·2% (10·1–10·4%)	166 724 (7·8%)	5·4% (5·3–5·6%)	14·8 (13·0–16·8)
History of adversity	58 107 (2·7%)	3274 (3269–3279)	9·0% (8·7–9·2%)	9·5% (9·2–9·7%)	9·1% (8·9–9·3%)	10·1% (9·9–10·4%)	56 944 (2·7%)	5·9% (5·7–6·1%)	17·2 (14·0–21·0)
History of mental health or behavioural conditions	51 312 (2·4%)	3237 (3231–3242)	10·0% (9·7–10·2%)	10·9% (10·6–11·2%)	9·2% (8·9–9·4%)	10·2% (9·9–10·4%)	50 166 (2·4%)	6·0% (5·8–6·2%)	18·7 (15·1–22·9)
Most deprivation†	580 631 (27·2%)	3304 (3302–3305)	9·6% (9·3–9·9%)	7·2% (7·1–7·2%)	9·1% (9·1–9·2%)	9·2% (9·1–9·3%)	577 357 (27·1%)	4·2% (4·2–4·3%)	12·4 (11·5–13·3)
No risk factors	1 377 706 (64·5%)	3408 (3406–3409)	4·8% (4·7–4·8%)	5·9% (5·9–5·9%)	6·3% (6·3–6·4%)	11·1% (11·1–11·2%)	1 374 650 (64·6%)	3·2% (3·1–3·2%)	6·2 (5·8–6·7)

Data are n (%), mean (95% CI), % (95% CI), or n per 100 000 infants (95% CI). Psychosocial risk factors were identified in the 2 years before 20 weeks of pregnancy.

* Small (<10th percentile of birthweight for gestation) or large (>90th percentile of birthweight for gestation) for gestation, derived from national birthweight percentiles.

† Most deprived quintile of the Index of Multiple Deprivation.

**Figure 3:**
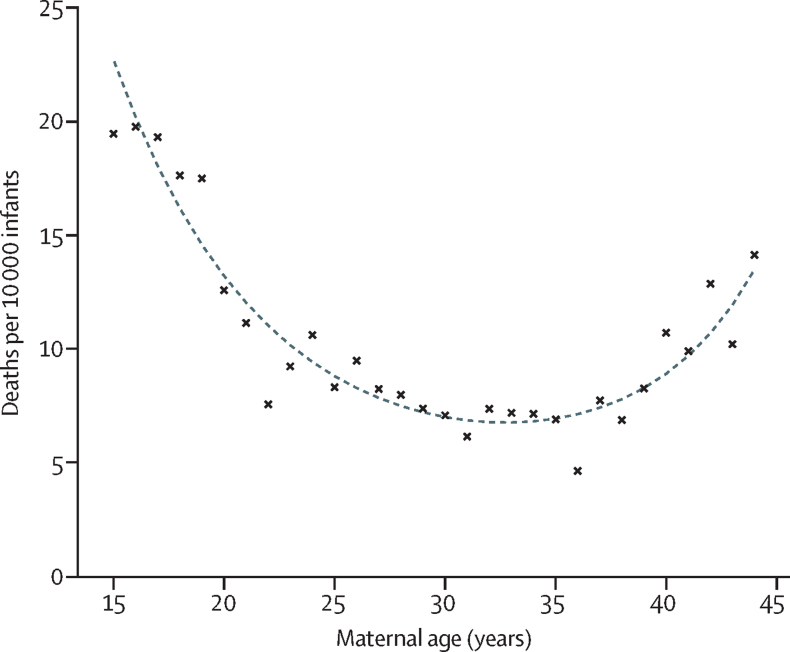
Crude (unadjusted) association between maternal age and infant mortality in the 12 months from postnatal discharge

Post-discharge mortality rates followed a U-shaped curve, with lowest rates observed for mothers aged 30–35 years (figure 3). Infants born to teenage mothers had the highest mortality rates, corresponding to an additional 10·2 deaths (95% CI 7·5–12·9) per 10 000 infants compared with mothers aged 20–44 years (appendix p 9). Those born to mothers with a history of adversity or mental health or behavioural conditions were also at increased risk of mortality (figure 2; appendix p 9). The highest PAF for mortality was for women living in the most deprived areas: 13·5% (9·4–17·5) of deaths were attributable to deprivation (table 1).

Results of the sensitivity analysis using complete case analysis were almost identical to those from the primary analysis using multiple imputation (appendix p 10).

## Discussion

Our population-based cohort study fills an evidence gap by examining which women should be considered at high risk for adverse birth and infant outcomes on the basis of multiple psychosocial risk factors recorded routinely in hospital records during or before pregnancy. All of the psychosocial risk factors (current or previous teenage birth, a history of adversity or mental health or behavioural conditions, or living in the most deprived areas) were associated with the adverse infant outcomes of low birthweight, unplanned admission for injury, and post-discharge mortality. The effect of these risk factors was consistently high across all maternal age groups. Overall, 36% of women had at least one risk factor, and although teenage mothers were at highest risk of poor infant outcomes, the majority (88%) of women with at least one risk factor were older than 20 years at delivery.

A major strength of our study is that we considered multiple psychosocial risk factors and outcomes, using national data linking over 2 million delivery and birth records. Without this linkage, it would not have been possible to evaluate how maternal exposures before pregnancy influence infant outcomes.^22^ A further strength is that outcomes were based on national policy priorities and that effect sizes were similar to those for other well recognised risk factors for poor infant outcomes.^2^, ^8^ For example, the crude risk differences in birthweight observed in this study were similar to those seen when comparing smoking and non-smoking mothers (150–200 g) and relative risks were comparable to those identified for parental mental health and infant injuries (1·3).^11^, ^12^ By considering and adjusting for a range of psychosocial risk factors within a national cohort, our study fills a gap in evidence on which factors are associated with the greatest population attributable risk.

Our study was limited by definitions of mental health or behavioural conditions and adversity that were based on hospital data: we only captured cases that were severe enough to be recorded in a hospital admission record. However, we included up to 20 diagnostic codes, and so would have captured admissions related to these conditions, even when these conditions were not the primary cause of admission. We did not have information on smoking or body-mass index, on the amount of support families received (eg, number of home visits by midwives or health visitors, participation in the Family Nurse Partnership [FNP], teenage pregnancy units, and housing or other services), or on childhood developmental outcomes. However, only up to a quarter of the 3·5% of first time teenage mothers in our study would have received FNP during this period, and any benefits of this intervention are likely to have led to an underestimation of the effects of psychosocial risk factors. We did not capture stillbirths or miscarriages, which might also have underestimated the effect of psychosocial risk factors.^30^ Our study included only singleton births, and we excluded mothers either younger than 15 years or older than 44 years. Future research could consider other relevant outcomes (eg, presentations to emergency departments) and explore associations for the 12% of births to mothers with multiple deliveries during the study period, which were excluded in this study. Evaluating regional variation in risk factors and outcomes could also be informative for policy. Our findings apply to England, but could be generalisable to other countries with similar maternal risk factors.

Proportionate universalism—the resourcing and delivering of services that are universal but have a scale and intensity proportionate to the level of disadvantage—is key to reducing health inequalities.^9^ For this strategy to work effectively, susceptible groups need to be identified early, on the basis of disease burden or determinants of health.^31^ We show that routine hospital data can inform who to target and provide an approach for quantifying the numbers of women at high risk.^20^ Presentations to hospital for emergency care for mental health or behavioural conditions, or adversity, before 20 weeks of pregnancy, provide important opportunities for interventions before pregnancy, including support for reproductive choice (eg, ensuring timely access to contraception) and preconception health (to improve birth outcomes).^5^ Such presentations during and after pregnancy should also prompt emergency care services to involve primary care and other community services to support the mother and child to improve child outcomes, for example through access to mental health care or intensive home visiting to build parenting capacity, ensure child safety, mitigate the effects of adversity, and help prepare for subsequent pregnancies. However, providing a universal service remains important for identifying women with needs not meeting thresholds for admission. Improved data collection and completeness of risk factor recording during antenatal visits and health visiting contacts, and improved data sharing and linkage (across health, social care, and education services) could support antenatal and health visiting services in making judgements for individual families and strengthen referral pathways between community and health-care services. Such linkage would also facilitate research on uptake and the wider societal benefits and costs of early interventions, and the opportunity costs of increased targeting schemes, in terms of diverting services from the universal model.

A further requirement for proportionate universalism is effective interventions. Interventions and specialised services that are offered to women vary across England.^32^ The FNP, which is targeted at first time teenage mothers, is one of the only programmes specifically recommended within the Healthy Child Programme.^8^ However, the FNP only targets first time teenage mothers (3·5% of this study population), whereas the highest risk of low birthweight and preterm birth in our study was seen in women with a history of mental health or behavioural conditions or adversity (who account for 4·0% of women across all age groups). Health visiting as an intervention for birth outcomes is too late: more evidence is needed on the propensity for these women to benefit from interventions before and during pregnancy, including whether different models of care are needed for younger versus older women with psychosocial risk factors.^33^, ^34^

Our study provides evidence to support health services planning, and evidence to support decisions about whether and how scarce universal public health services should be targeted, in the context of decreasing funding for public health services and the workforce (including health visiting).^35^ In terms of population attributable risk, deprivation was a key factor, followed by teenage pregnancy (11·3% of women were current or previous teenage mothers). Similar to previous studies, these effects were not fully explained by adjusting for other risk factors.^17^ Our findings indicate that primary prevention strategies could be targeted at the population level, on the basis of age and socioeconomic background. Such strategies need to integrate cross-sectoral agencies; this integration has for example been done by the multifaceted policy intervention to reduce teenage pregnancies in England (the Teenage Pregnancy Strategy), which involved health and education agencies and both contributed to a decline in teenage births and attenuated the steep deprivation gradient.^13^ Previous research has also shown that the effects of teenage motherhood persist for previous teenage mothers giving birth again in their 20s, meaning that strategies to improve reproductive choices for young women about the timing of their pregnancies could have lasting effects.^36^ There is a need to understand how existing services (eg, for teenage pregnancies, contraception, sexual health services, and drug and alcohol services) can best be integrated with support in early years to address the needs of women affected by psychosocial risk factors.

Our results also support previous evidence showing that having a history of admissions for mental health or behavioural conditions or adversity is an important potential risk indicator for poor infant outcomes that should be considered when supporting individual women in clinical practice.^18^ A UK study using primary care records showed that a majority of mothers registered with a general practice had received mental health treatment or diagnosis between the birth of their child and the child's 16th birthday.^37^ Parental mental health is strongly linked to child and adolescent mental health and mortality in early adulthood.^38^, ^39^ The high prevalence of these parental health problems points to the need for services in primary care, mental health, and maternity and child health to be more responsive to the needs of parents to improve outcomes for parents and their children.

Our findings show disparities in adverse outcomes for the 36% of women with one or more of the psychosocial risk factors measured in our study. Given that our study was not able to account for mental health problems not recorded in hospital admission records and that we only considered the highest quintile of deprivation, our results are probably an underestimate of the true burden of adverse infant outcomes in the community. Effective interventions before, during, and after pregnancy are needed to reduce the downstream burden on health services and prevent long-term adverse effects for children. Upstream, public health, and economic strategies are important to reduce socioeconomic disparities. Within health care, efforts are needed across primary and secondary services to address potential effects of psychosocial presentations among women on pregnancy and child outcomes.

## Data sharing

We are unable to share the individual data used for this study. HES data can be requested through NHS Digital.
