# Supplementary material for: Associations between pre-pregnancy psychosocial risk factors and infant outcomes: a population-based cohort study in England
Source: Lancet Public Health. 2021 Jan 28;6(2):e97–e105. doi: 10.1016/S2468-2667(20)30210-3 (PMC7848754; doi:10.1016/S2468-2667(20)30210-3)
Supplement: Supplementary appendix [file mmc1.pdf]

THE LANCET
Public Health

Supplementary appendix

This appendix formed part of the original submission and has been peer reviewed. We post it as supplied by the authors.

Supplement to: Harron K, Gilbert R, Fagg J, Guttmann A, van der Meulen J. Associations between pre-pregnancy psychosocial risk factors and infant outcomes: a population-based cohort study in England. *Lancet Public Health* 2021; **6:** e97–105.

Appendix

Appendix Figure 1: Creation of the analysis cohort

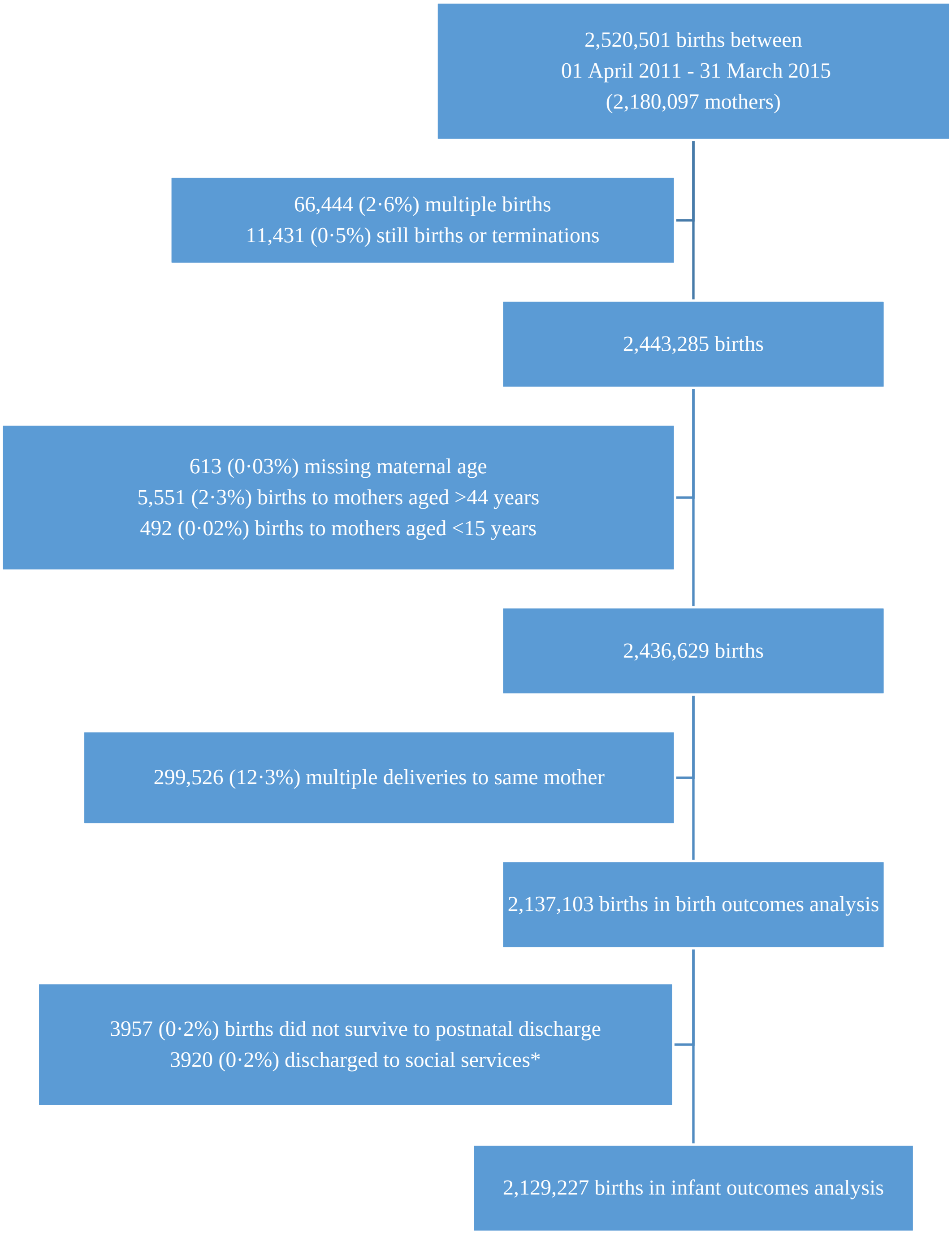

* Local Authority residential accommodation (i.e. where care is provided) or foster care, according to ‘destination on discharge’ variable in HES

Appendix Figure 2: Relationship between maternal age and psychosocial risk-factors identified in the two years prior to 20 weeks of pregnancy.

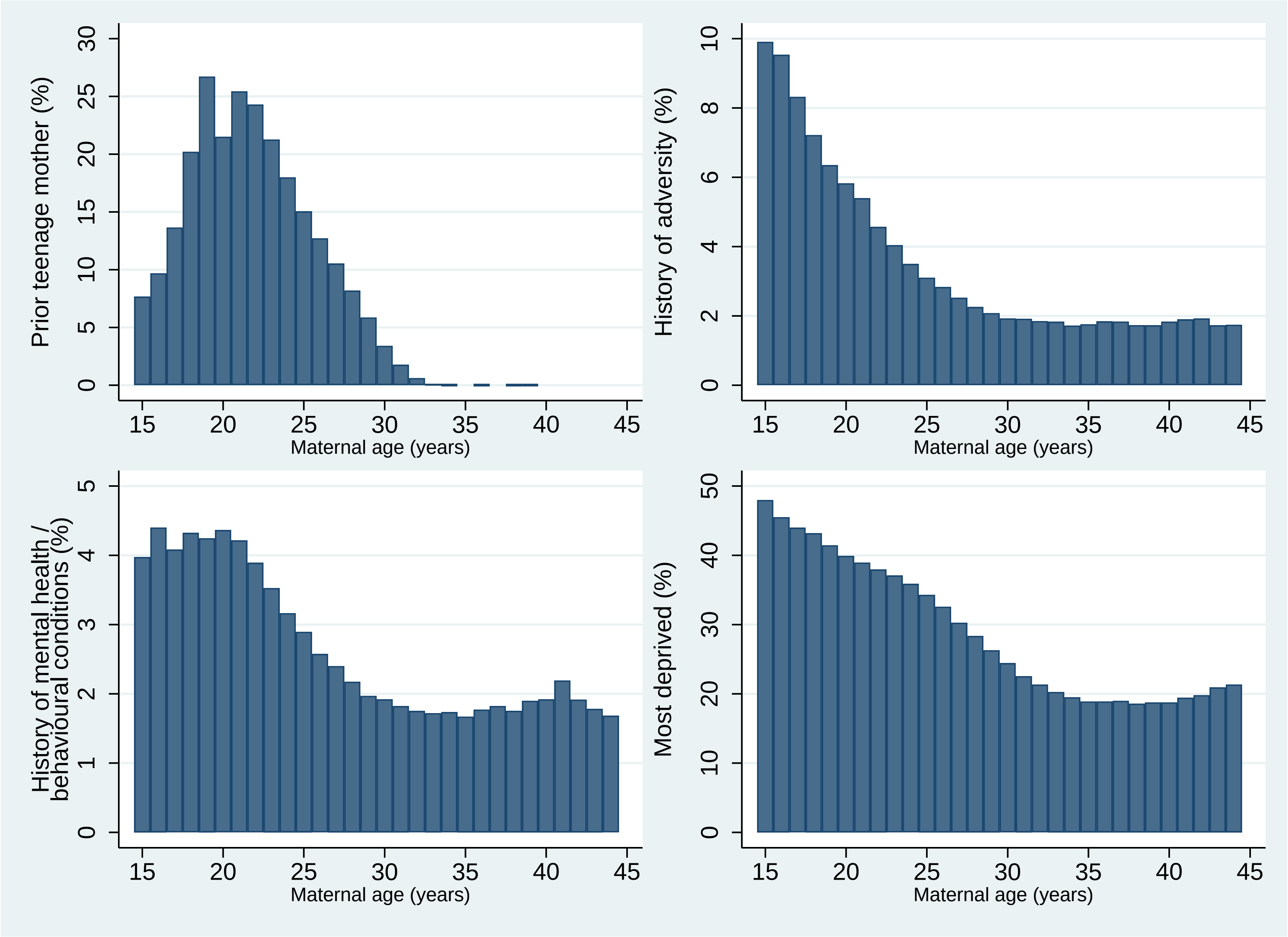

Appendix Table 1: ICD 10 code lists for risk-factors relating to adversity and mental health / behavioural conditions.

Code lists were derived from the following studies:

Herbert A, Gilbert R, González-Izquierdo A, et al. Violence, self-harm and drug or alcohol misuse in adolescents admitted to hospitals in England for injury: a retrospective cohort study. BMJ Open. 2015;5(2):e006079.

Hardelid P, Dattani N, Gilbert R. Estimating the prevalence of chronic conditions in children who die in England, Scotland and Wales: a data linkage cohort study. BMJ Open. 2014;4(8).

Group	Description	ICD10 Code
**Adversity-related admissions**		
Violence	Maltreatment syndromes	T74
	Effects of other deprivation (extreme neglect)	T73
	Perpetrator of neglect and other maltreatment syndromes	Y06, Y07
	Assault by bodily force and sexual assault	Y04, Y05
	Other types of assault	X85-Y03, Y08-Y09
	Events of undetermined intent	Y20-Y34
	Examination and observation following other inflicted injury	Z04.5
	Examination and observation for other reasons: request for expert evidence	Z04.8
Self-harm	Sequelae of intentional self-harm	Y87
	Intentional self-poisoning by and exposure to … drugs	X60-X63
	…other and unspecified drugs, medicaments and biological substances	X64
	…alcohol	X65
	…organic solvents and halogenated hydrocarbons and their vapours	X66
	…other gases and vapours	X67
	…pesticides	X68
	…other and unspecified chemicals and noxious substances	X69
	Intentional self-harm by… hanging, strangulation and suffocation	X70
	…drowning and submersion	X71
	…firearm discharge	X72-X74
	…explosive material	X75

	…smoke, fire and flames, or steam, hot vapours and hot objects	X76-X77
	…sharp/blunt objects	X78-X79
	...jumping from a high place	X80
	…jumping or lying before a moving object, or crashing a motor vehicle	X81-82
	…other specified means	X83
	…unspecified means	X84
	Personal history of self-harm	Z91.5
Substance misuse	Mental and behavioural disorders due to psychoactive substance use	F11-F17, F19 (not F17.1)
	Finding of drugs not normally found in blood	R78.1-R78.5
	Poisoning by drugs, medicaments and biological substances	T36-T50 (not T50.6)
	Poisoning, undetermined intent	Y10-Y14
	Drug rehabilitation	Z50.3
	Drug abuse counselling and surveillance	Z71.5
	Drug use	Z72.2
	Personal history of psychoactive substance abuse	Z864
	Mental and behavioural disorders due to use of volatile solvents	F18
	Accidental poisoning by and exposure to noxious substances	X40–X44, X46-X49
	Poisoning by chemical or noxious substance, undetermined intent	Y16-Y19
	Special epileptic syndromes - (related to alcohol, drugs, etc.)	G40.5
	Blood-alcohol and blood-drug test	Z04.0
	Alcohol-induced pseudo-Cushing's syndrome	E24.4
	Mental and behavioural disorders due to use of alcohol	F10
	Degeneration of nervous system due to alcohol	G31.2
	Alcoholic polyneuropathy	G62.1
	Alcoholic myopathy	G72.1
	Alcoholic cardiomyopathy	I42.6
	Alcoholic gastritis	K29.2
	Alcoholic liver disease	K70
	Alcohol-induced acute pancreatitis	K85.2
	Alcohol-induced chronic pancreatitis	K86.0
	Maternal care for (suspected) damage to fetus from alcohol	O35.4

	Finding of alcohol in blood	R78.0
	Poisoning: antidotes and chelating agents, not elsewhere classified	T50.6
	Toxic effect of alcohol	T51
	Accidental poisoning by exposure to alcohol	X45
	Poisoning by exposure to alcohol, undetermined intent	Y15
	Evidence of alcohol involvement determined by blood alcohol level	Y90
	Evidence of alcohol involvement determined by level of intoxication	Y91
	Alcohol rehabilitation	Z50.2
	Alcohol abuse counselling and surveillance	Z71.4
	Alcohol use	Z72.1
**Mental health conditions / behavioural disorders***		
	Organic, including symptomatic, mental disorders	F00-F09
	Schizophrenia, schizotypal and delusional disorders	F20-F29
	Mood [affective] disorders	F30-F39
	Neurotic, stress-related and somatoform disorders	F40-F48
	Behavioural syndromes associated with physiological disturbances and physical factors	F50-F59
	Disorders of adult personality and behaviour	F60-F69
	Mental retardation	F70-F79
	Disorders of psychological development	F80-F89
	Behavioural and emotional disorders with onset usually occurring in childhood and adolescence	F90-F98
	Unspecified mental disorder	F99
	Sedatives, hypnotics and antianxiety drugs	Y47
	Psychotropic drugs, not elsewhere classified	Y49
	Personal history of other mental and behavioural disorders	Z865

* Excluding those that fall under adversity (e.g. relating to self-harm or substance misuse)

Appendix Table 2: Code lists for risk-factors relating to pregnancy, delivery and birth.

Code lists were derived from the following study:

Harron K, Gilbert R, Cromwell D, et al. International comparison of emergency hospital use for infants: data linkage cohort study in Ontario and England. BMJ Qual Saf. 2018;27(1):31-9.

Group	Type	Description	ICD10 codes
Pregnancy risk-factor	ICD10 codes	Intrauterine fetal death<br>Eclampsia<br>Gestational hypertension<br>Placental abruption or infarction<br>Uterine rupture<br>Diabetes in pregnancy	O364, P95<br>O14,O15<br>O13,O16<br>O45,O431, O438, O439<br>O710, O711<br>P700, O24, E10-E14
Delivery risk-factor	ICD10 codes	Birth trauma<br>Complications of delivery<br>Hypoxia<br>Amniotic fluid embolism<br>Chorioamnionitis<br>Umbilical cord problem<br>Fetal hemorrhage<br>Maternal hemorrhage<br>Umbilical cord prolapse	P10-P15<br>P03<br>P20-P21<br>O881<br>O411 P027-P029<br>P020, P024-P026<br>P50, P51, P53, P54<br>O430<br>O69
Neonatal medical condition	ICD10 codes	Congenital anomalies	Q00 Q01 Q02 Q03 Q04 Q05 Q06 Q07 Q20 Q212 Q213 Q214 Q218 Q219 Q22 Q23 Q24 Q251 Q252 Q253 Q254 Q255 Q256 Q257 Q258 Q259 Q26 Q282 Q283 Q289 Q30 Q31 Q32 Q33 Q34 Q390 Q391 Q392 Q393 Q394 Q41 Q42 Q43 Q44 Q45 Q60 Q61 Q62 Q63 Q64 Q722 Q750 Q752 Q759 Q760 Q761 Q762 Q764 Q765 Q766 Q767 Q77 Q780 Q781 Q782 Q783 Q784 Q788 Q789 Q790 Q791 Q792 Q793 Q794 Q795 Q799 Q81 Q851 Q871 Q872 Q873 Q874 Q878 Q879 Q897 Q899 Q909 Q913 Q914 Q917 Q928 Q93 Q950 Q969 Q97 Q98 Q992 Q998 Q999
	ICD10 codes	Complex chronic conditions	B20-B23, D55, D561, D562, D570-D572, D58, D80-D84, D898, D899, E343, E70-E730, E74, E76-E79, E803-E807, E83-E85, E881, E882, E888, E889, F70, F72, F73, F842, G10-G12, G20, G23, G240-G242, G248, G250-G256, G318, G319, G40, G41, G71, G72, G80-G82, G901, G903, G904, G91, G940-G942, G95, G99, I42, I44, I45, I47-I49, I515, K44, K50-K51, K73-K74, K754, K758-K760, M41, N18, P27, P90
Delivery by caesarean section	OPCS codes	Elective caesarean delivery<br>Other caesarean delivery	R17<br>R18
	ICD10 codes	Single delivery by caesarean section<br>Multiple delivery, all by caesarean section<br>Fetus and newborn affected by caesarean delivery	O82<br>O842<br>P034

Appendix Table 3: Characteristics of study population: relationship between maternal age and psychosocial risk-factors identified in the two years prior to 20 weeks of pregnancy.

			Maternal age (years)									
	Total		15-19		20-24		25-29		30-34		35-44	
	N	%	N	%	N	%	N	%	N	%	N	%
**Total (row %)**	2,137,103 (100·0)		93,279 (4·4)		365,908 (17·1)		598,484 (28·0)		644,083 (30·1)		435,349 (20·4)	
**Any risk-factor***	759,397	35·5	93,279	100·0	190,707	52·1	225,778	37·7	157,344	24·4	92,289	21·2
Any risk-factor excluding deprivation	306,208	14·3	93,279	100·0	94,829	25·9	78,840	13·2	26,300	4·1	12,960	3·0
**Prior teenage mother**	168,186	7·9	19,529	20·9	79,712	21·8	60,984	10·2	7957	1·2	<5	0·0
**History of adversity related admissions**	58,107	2·7	6765	7·3	16,453	4·5	15,123	2·5	11,940	1·9	7826	1·8
**History of admissions for mental health or behavioural conditions**	51,312	2·4	3960	4·3	13,705	3·8	14,220	2·4	11,558	1·8	7869	1·8
History of adversity OR mental health / behavioural conditions	85,811	4·0	7903	8·5	22,761	6·2	23,168	3·9	19,022	3·0	12,957	3·0
**National quintile of deprivation**												
Least deprived	320,896	15·0	6009	6·4	29,757	8·1	71,843	12·0	118943	18·5	94,344	21·7
2	341,761	16·0	9146	9·8	42,476	11·6	87,164	14·6	117941	18·3	85,034	19·5
3	396,818	18·6	14,271	15·3	61,968	16·9	111,592	18·6	125772	19·5	83,215	19·1
4	480,889	22·5	23,397	25·1	91,707	25·1	143,823	24·0	136684	21·2	85,278	19·6
Most deprived	580,631	27·2	39,949	42·8	137,872	37·7	180,244	30·1	139905	21·7	82,661	19·0
Missing	16,108	0·8	507	0·5	2128	0·6	3818	0·6	4838	0·8	4817	1·1
**Primiparous**	877,431	41·1	73,750	79·1	192,359	52·6	253,910	42·4	234808	36·5	122,604	28·2
**Ethnicity**												
White	1,510,146	70·7	76,252	81·7	275,796	75·4	411,518	68·8	444,667	69·0	301,913	69·3
Mixed	98,991	4·6	5166	5·5	17,596	4·8	24,304	4·1	28,179	4·4	22,746	5·2
Asian	225,149	10·5	1877	2·0	28,356	7·7	78,094	13·0	76,714	11·9	40,108	9·2
Black	99,589	4·7	2729	2·9	13,050	3·6	27,326	4·6	30,773	4·8	25,711	5·9
Other	64,187	3·0	1685	1·8	8748	2·4	18,066	3·0	20,615	3·2	15,073	3·5
Unknown	139,041	6·5	5570	6·0	22,362	6·1	38,176	6·4	43,135	6·7	29,798	6·8

*Current or previous teenage birth, history of adversity or mental health / behavioural conditions, or living in the most deprived areas

Appendix Table 4: Relationship between maternal age and psychosocial risk-factors identified within 1, 2 and 5 years prior to 20 weeks of pregnancy

			Maternal age (years)									
	**Total**		**15-19**		**20-24**		**25-29**		**30-34**		**35-44**	
	N	%	N	%	N	%	N	%	N	%	N	%
**Total (row %)**	2,137,103 (100%)		93,279 (4·4)		365,908 (17·1)		598,484 (28·0)		644,083 (30·1)		435,349 (20·4)	
**History of mental health or behavioural conditions**												
1 year look back period	26,967	1·3	2300	2·5	7456	2·0	7424	1·2	5865	0·9	3922	0·9
2 year look back period	51,312	2·4	3960	4·3	13,705	3·8	14,220	2·4	11,558	1·8	7869	1·8
5 year look back period	89,923	4·2	6177	6·6	22,715	6·2	25,663	4·3	20,867	3·2	14,501	3·3
**History of adversity**												
1 year look back period	29,049	1·4	3630	3·9	8365	2·3	7604	1·3	5727	0·9	3723	0·9
2 year look back period	58,107	2·7	6765	7·3	16,453	4·5	15,123	2·5	11,940	1·9	7826	1·8
5 year look back period	117,416	5·5	11,574	12·4	33,012	9·0	31,942	5·3	24,777	3·9	16,111	3·7

Appendix Table 5: Crude and adjusted^ risk differences and relative risks for birth weight, and unplanned injury admissions and mortality within 12 months of postnatal discharge. Comparisons are between mothers with each risk-factor identified in the two years prior to 20 weeks of pregnancy, and mothers without that specific risk-factor.

		**Risk difference (95% CI)**		**Relative risk$ (95% CI)**	
Birth outcomes (N = 2,137,103)		Crude	Adjusted	Crude	Adjusted
Birth weight (grams)	Teenage mothers	128·9 (121·4, 136·4)	91·4 (85·5, 97·4)	1·46 (1·40, 1·51)	1·24 (1·19, 1·28)
	Prior teenage mothers	38·3 (32·2, 44·4)	65·6 (60·1, 71·0)	1·20 (1·17, 1·24)	1·33 (1·30, 1·37)
	History of adversity	101·8 (89·7, 113·9)	65·0 (54·6, 75·4)	1·66 (1·58, 1·74)	1·35 (1·29, 1·42)
	History of mental health / behavioural conditions	135·7 (124·4, 147·1)	124·2 (114·2, 134·2)	1·83 (1·75, 1·92)	1·63 (1·56, 1·70)
	Most deprived	97·4 (87·4, 107·5)	62·0 (56·9, 67·1)	1·43 (1·39, 1·48)	1·32 (1·28, 1·36)
Outcomes in 12 months from postnatal discharge (N=2,129,227)					
Unplanned injury admissions+ (%)	Teenage mothers	3·7 (3·4, 4·0)	3·6 (3·3, 3·9)	2·20 (2·11, 2·29)	2·18 (2·09, 2·27)
	Prior teenage mothers	2·0 (1·9, 2·2)	0·6 (0·5, 0·8)	1·57 (1·53, 1·62)	1·09 (1·06, 1·12)
	History of adversity	1·4 (2·2, 2·7)	1·1 (0·9, 1·3)	1·66 (1·60, 1·73)	1·24 (1·19, 1·28)
	History of mental health / behavioural conditions	2·5 (2·3, 2·8)	1·4 (1·2, 1·7)	1·69 (1·63, 1·76)	1·32 (1·27, 1·38)
	Most deprived	0·8 (0·5, 1·1)	0·5 (0·2, 0·7)	1·24 (1·16, 1·32)	1·14 (1·07, 1·20)
Post-discharge mortality (per 10,000 infants)	Teenage mothers	11·1 (8·6, 13·6)	10·2 (7·5, 12·9)	2·59 (2·20, 3·05)	2·68 (2·21, 3·27)
	Prior teenage mothers	6·9 (5·2, 8·6)	3·3 (1·3, 5·2)	1·87 (1·65, 2·13)	1·22 (1·03, 1·44)
	History of adversity	9·0 (5·8, 12·3)	4·3 (0·9, 7·7)	2·10 (1·72, 2·56)	1·39 (1·08, 1·79)
	History of mental health / behavioural conditions	10·5 (6·9, 14·2)	6·5 (2·5, 10·5)	2·29 (1·87, 2·80)	1·68 (1·29, 2·18)
	Most deprived	5·4 (4·2, 6·6)	3·7 (2·5, 4·9)	1·77 (1·59, 1·97)	1·49 (1·33, 1·68)

^Adjusted for all maternal risk-factors, ethnic group, and parity. $Relative risk for birthweight is for low birth weight (<2500g). +Including mortality

Appendix Table 6: Sensitivity analysis using complete case analysis: Crude and adjusted^ risk differences and relative risks for birth weight, and unplanned injury admissions and mortality within 12 months of postnatal discharge. Comparisons are between mothers with each risk-factor identified in the two years prior to 20 weeks of pregnancy, and mothers without that specific risk-factor.

		Risk difference (95% CI)		Relative risk$ (95% CI)	
Birth outcomes (N = 2,137,103)		Crude	Adjusted	Crude	Adjusted
Birth weight (grams)	Teenage mothers	129·5 (122·0, 137·1)	89·0 (82·9, 95·1)	1·46 (1·40, 1·52)	1·21 (1·17, 1·26)
	Prior teenage mothers	39·4 (33·1, 45·8)	63·6 (57·9, 69·3)	1·22 (1·19, 1·25)	1·32 (1·29, 1·36)
	History of adversity	102·4 (91·9, 112·8)	61·9 (53·0, 70·8)	1·65 (1·58, 1·71)	1·34 (1·28, 1·39)
	History of mental health / behavioural conditions	140·7 (131·8, 149.6)	128·6 (121·1, 136·2)	1·83 (1·77, 1·89)	1·62 (1·56, 1·68)
	Most deprived	96·4 (86·2, 106·5)	60·4 (55·0, 65·8)	1·43 (1·39, 1·48)	1·32 (1·28, 1·36)
Outcomes in 12 months from postnatal discharge (N=2,129,227)					
Unplanned injury admissions+ (%)	Teenage mothers	3·7 (3·4, 4·0)	3·6 (3·3, 3·9)	2·20 (2·11, 2·29)	2·18 (2·09, 2·27)
	Prior teenage mothers	2·0 (1·9, 2·2)	0·6 (0·5, 0·8)	1·57 (1·53, 1·62)	1·09 (1·06, 1·12)
	History of adversity	2·4 (2·2, 2·7)	1·1 (0·9, 1·3)	1·66 (1·60, 1·73)	1·24 (1·19, 1·28)
	History of mental health / behavioural conditions	2·5 (2·3, 2·8)	1·4 (1·2, 1·7)	1·69 (1·63, 1·76)	1·32 (1·27, 1·38)
	Most deprived	0·9 (0·6, 1·2)	0·5 (0·3, 0·8)	0·76 (1·16, 1·33)	1·14 (1·08, 1·21)
Post-discharge mortality (per 10,000 infants)	Teenage mothers	11·1 (8·6, 13·6)	10·2 (7·5, 13·3)	2·59 (2·20, 3·05)	2·69 (2·21, 3·27)
	Prior teenage mothers	6·9 (5·2, 8·6)	3·3 (1·3, 5·3)	1·96 (1·73, 2·23)	1·22 (1·03, 1·44)
	History of adversity	9·0 (5·8, 12·3)	4·3 (0·9, 7·7)	2·20 (1·80, 2·69)	1·39 (1·08, 1·79)
	History of mental health / behavioural conditions	10·5 (6·9, 14·2)	6·5 (2·5, 10·5)	2·39 (1·96, 2·92)	1·68 (1·29, 2·18)
	Most deprived	5·4 (4·2, 6·6)	3·7 (2·5, 4·9)	1·70 (1·52, 1·91)	1·49 (1·32, 1·68)

^Adjusted for all maternal risk-factors, ethnic group, and parity. $Relative risk for birthweight is for low birth weight (<2500g). +Including mortality.
